# Insights into immune responses in oral cancer through proteomic analysis of saliva and salivary extracellular vesicles

**DOI:** 10.1038/srep16305

**Published:** 2015-11-05

**Authors:** Flavia V. Winck, Ana Carolina Prado Ribeiro, Romênia Ramos Domingues, Liu Yi Ling, Diego Mauricio Riaño-Pachón, César Rivera, Thaís Bianca Brandão, Adriele Ferreira Gouvea, Alan Roger Santos-Silva, Ricardo D. Coletta, Adriana F. Paes Leme

**Affiliations:** 1 Laboratório de Espectrometria de Massas, Laboratório Nacional de Biociências, LNBio, CNPEM, Campinas, SP, Brazil; 2 Instituto do Câncer do Estado de São Paulo, Octavio Frias de Oliveira, ICESP, São Paulo, SP, Brazil; 3 Laboratório Nacional de Ciência e Tecnologia do Bioetanol, CTBE, CNPEM, Campinas, SP, Brazil; 4 Faculdade de Odontologia de Piracicaba, Universidade Estadual de Campinas, UNICAMP, Piracicaba, SP, Brazil; 5 Departamento de Ciencias Básicas Biomédicas, Universidad de Talca (UTALCA), Talca, Chile

## Abstract

The development and progression of oral cavity squamous cell carcinoma (OSCC) involves complex cellular mechanisms that contribute to the low five-year survival rate of approximately 20% among diagnosed patients. However, the biological processes essential to tumor progression are not completely understood. Therefore, detecting alterations in the salivary proteome may assist in elucidating the cellular mechanisms modulated in OSCC and improve the clinical prognosis of the disease. The proteome of whole saliva and salivary extracellular vesicles (EVs) from patients with OSCC and healthy individuals were analyzed by LC-MS/MS and label-free protein quantification. Proteome data analysis was performed using statistical, machine learning and feature selection methods with additional functional annotation. Biological processes related to immune responses, peptidase inhibitor activity, iron coordination and protease binding were overrepresented in the group of differentially expressed proteins. Proteins related to the inflammatory system, transport of metals and cellular growth and proliferation were identified in the proteome of salivary EVs. The proteomics data were robust and could classify OSCC with 90% accuracy. The saliva proteome analysis revealed that immune processes are related to the presence of OSCC and indicate that proteomics data can contribute to determining OSCC prognosis.

Oral cancer represents 1 to 2% of all types of cancer worldwide, and oral squamous cell carcinoma (OSCC) is the most frequent histopathological type reported among patients^1^. This disease can affect different sites in the oral cavity, such as the tongue, floor of the mouth and cheeks, and smoking and alcohol ingestion is responsible for approximately 90% of OSCC cases^2^,^3^,^4^. Half of the patients with oral cancer are diagnosed only when the disease has reached an advanced clinical stage (III/IV), leading to a five-year survival rate of only 20% in these cases^1^,^5^,^6^. OSCC prognosis is currently based on the clinical staging system of tumor-lymph node-metastasis of the disease (TNM system). However, this system is not optimal because tumors may present distinct biological characteristics even at similar developmental stages^7^. Thus, the identification of additional parameters or biological markers that assist with determining the prognosis of patients with OSCC is essential.

One promising strategy for the discovery of new biomarkers consists of the identification of the protein profile of body fluids, such as saliva, which may be used to characterize a specific disease. The repertoire of disease-related proteins and other molecules can be identified by mass spectrometry. Although the use of saliva is not novel, this approach is receiving great interest as a diagnostic fluid because harvesting saliva is performed using non-invasive methods. Previous studies reporting the proteomic analysis of saliva from healthy individuals have already indicated the potential of this approach for monitoring health^8^. Furthermore, recent studies have shown that saliva contains many signaling molecules that may be indicative of cancer^9^,^10^.

Moreover, cancer cells can generate several types of extracellular vesicles, ranging from 30 nm to a few micrometers in diameter, which may be shed from the cytoplasmic membrane^11^. These extracellular vesicles (EVs) can deliver molecules (such as proteins, mRNA, microRNA, rRNA, tRNA, DNA and lipids) that have been suggested to participate in important intracellular signaling mechanisms even in distant target cells^11^,^12^,^13^. Although many potential biomarkers for oral cancer have been identified in human saliva, the role of such molecules in oral cancer is not completely understood^10^,^14^,^15^,^16^. Several proteins, including CD44, IL-6, IL-8 and defensin-1, have been suggested to be OSCC biomarkers. The expression of these molecules has also been reported in other types of cancer, indicating that common cancer cell responses may underlie tumor progression^17^,^18^,^19^,^20^. Furthermore, global similarities between the differing types of cancer indicate that cancer complexity may reside in the cellular host responses, which could indeed influence the mechanisms by which cancer progresses in different individuals.

In the present study, we examined the proteome profile of whole saliva and salivary EVs from patients with oral cancer (patients with and without tumor lesions) and healthy individuals by mass spectrometry analysis. Due to significant differences in processes related to inflammatory and humoral immune responses, peptidase inhibitor activity, iron coordination and protease binding, the two classes of individuals (healthy vs. OSCC) were distinguished with 90% accuracy based on the proteomics data. Moreover, proteome functional annotation revealed that differentially expressed salivary proteins may indeed be related to cell-to-cell signaling and cellular interaction mechanisms.

## Results

### Differentially expressed proteins in whole saliva from oral cancer patients showed potential roles in peptidase regulation and immune responses

**Figure 1:**
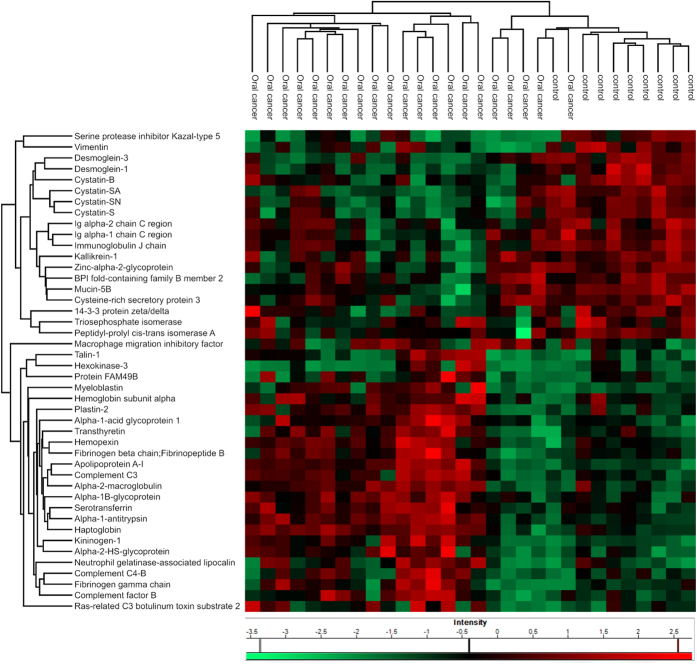
Differentially expressed proteins in whole saliva from human oral cancer. LFQ intensity values of the salivary proteins identified from oral cancer patients were compared to the LFQ values of salivary proteins identified from healthy individuals, and proteins with a significant differential abundance were detected using the ANOVA (p < 0.05) method. Clustering analysis of the data was performed using a Euclidian distance method for the z-score normalized LFQ values. The z-score normalized values are shown in a heat map that represents the variation in protein abundance between the analyzed samples. Groups of oral cancer and healthy individuals and the corresponding protein names are indicated.

We analyzed the whole salivary proteome and the proteome from salivary EVs isolated from healthy individuals and oral cancer patients (“lesion” plus “no lesion” individuals). The clinical classifications of the patients are listed in Supplemental Table 1. Relative protein quantification was performed using the label-free quantification (LFQ) method, and our data showed high reproducibility (Supplemental Figure 1 and Supplemental Figure 2). This analysis resulted in the identification of 507 proteins after data pre-processing (excluding contaminants, reverse sequences and only identified by site) (Supplemental Table 2) from which 147 proteins had at least five valid LFQ intensity values in at least one group (healthy, n = 9, or oral cancer, n = 21). The filtered dataset containing 147 proteins was subjected to further data analysis using a one-way ANOVA test (p < 0.05) resulting in 44 proteins showing differential expression. In addition, ANOVA p-values corrected by Storey´s method are shown (Fig. 1, Supplemental Table 3). Most of these differentially expressed proteins showed reduced expression in oral cancer patients. Additionally, nineteen proteins were exclusively identified in the healthy group, and 247 proteins were exclusively identified in the oral cancer group.

**Figure 2:**
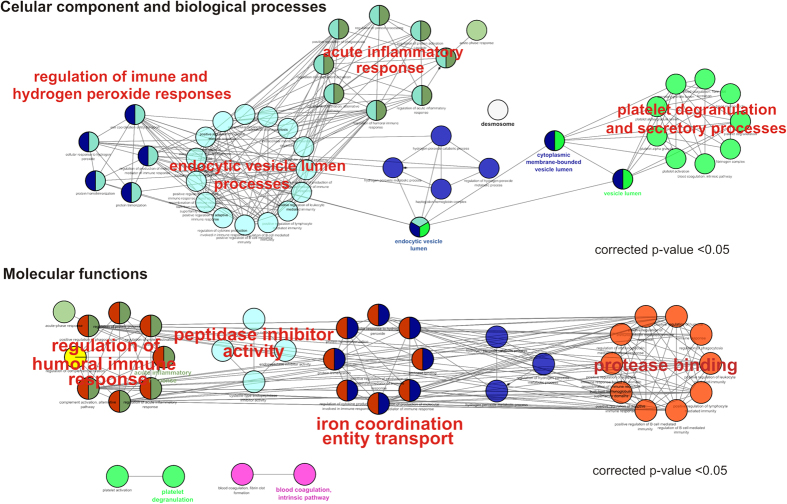
Network of overrepresented GO terms in the dataset of differentially expressed proteins in the whole saliva proteome. Proteins identified as differentially expressed in the whole saliva of patients with oral cancer and healthy individuals were subjected to a GO term enrichment analysis using the ClueGO plugin. Overrepresented GO terms (p-value < 0.05) for Biological Processes, Cellular Components and Molecular Function categories were visualized using the Cytoscape suite. The most significant GO terms within the dataset of significantly enriched terms are indicated in red font.

The results of GO enrichment analysis of the differentially expressed proteins (44 proteins, ANOVA p < 0.05) showed a significant overrepresentation of biological processes related to acute inflammatory response (p-value = 3.3E-07), regulation of humoral response (p-value = 3.1E-06) and regulation of hydrogen peroxide metabolic processes (p-value = 1.67E-04). Cellular components related to cytoplasmic membrane-bounded vesicle lumen (p-value = 5.9E-10) and endocytic vesicle lumen (p-value = 6.5E-06) components were overrepresented in the dataset. According to the IPA (Ingenuity® Systems, http://www.ingenuity.com) analysis, the molecular function cell-to-cell signaling and interaction was overrepresented in the dataset of differentially expressed proteins (Supplemental Table 4). Furthermore, overrepresented molecular function GO terms included humoral immune responses, peptidase inhibitor activity and protease binding (p-value < 0.05). A network of the overrepresented GO terms is shown in Fig. 2.

### Proteomic data analysis of whole saliva from healthy and oral cancer patients permitted classification of patients

Principal component analysis (PCA) was performed using the proteome LFQ data (no missing values) from whole saliva and indicated that groups of healthy individuals can be distinguished from the oral cancer individuals (Supplemental Figure 3). Therefore, we applied the support vector machines (SVM) method to the proteome dataset to evaluate whether the dataset could classify the two groups of individuals. Furthermore, proteins that could classify the two groups of individuals (healthy and OSCC) were identified through the application of the feature selection method. For the generation of the classification model using SVM, 571 attributes (protein intensity normalized by z-score) and 30 instances (individuals) were used, and a 3-fold cross-validation method was performed. Our classification model resulted in high precision (0.925), recall (0.9), and ROC (0.929) and a Kappa value of 0.78. The proteins identified as the best features for data classification are listed in Table 1 with their functional annotations.

### Oral cancer patients with lesions and without tumor lesions have differing proteome repertoires

**Figure 3:**
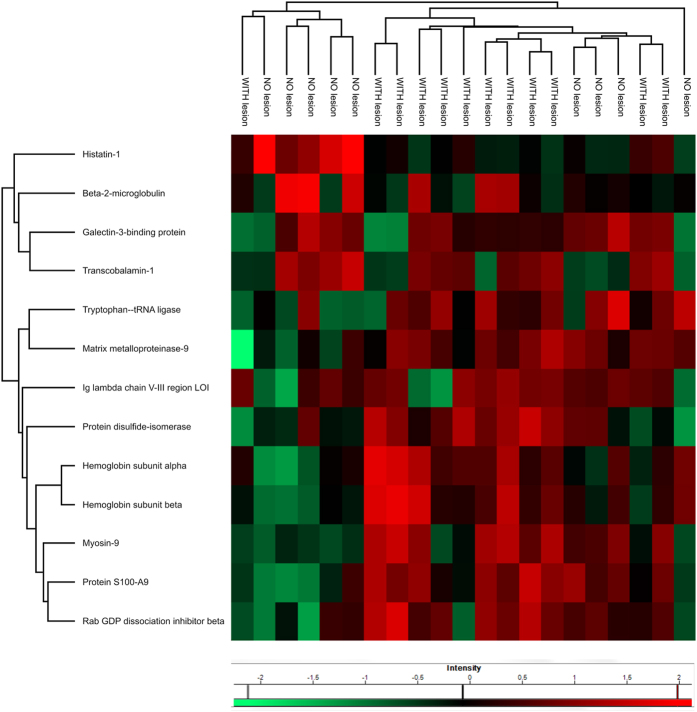
Differentially expressed proteins in response to the presence or absence of tumors in patients diagnosed with oral cancer. LFQ intensity values (log2) of the salivary proteins identified from diagnosed oral cancer patients with tumors (with lesion) were compared to patients without tumors (no lesion). Differentially expressed proteins were detected using the ANOVA method (p < 0.05). A Euclidean distance clustering analysis of these data was performed, and the differential protein abundance is shown in the heat map of normalized z-score values.

The influence of the presence or absence of tumor lesions in patients diagnosed with oral cancer was investigated. Among the total 507 proteins identified, 13 proteins were identified as differentially expressed between the “lesion” (n = 12) and “no lesion” (n = 9) groups (Fig. 3). A group of 38 proteins were identified exclusively in the saliva of patients with lesions, and 5 proteins were exclusively identified in the group of patients without lesions, considering proteins identified in at least two individuals. According to IPA knowledge base information, some of the differentially expressed proteins may play roles in processes related to cell movement, morphology and cell death (Supplemental Table 5).

### Proteomic profiling of salivary extracellular vesicles indicated overrepresentation of antigen binding and enzyme inhibitory functions

**Figure 4:**
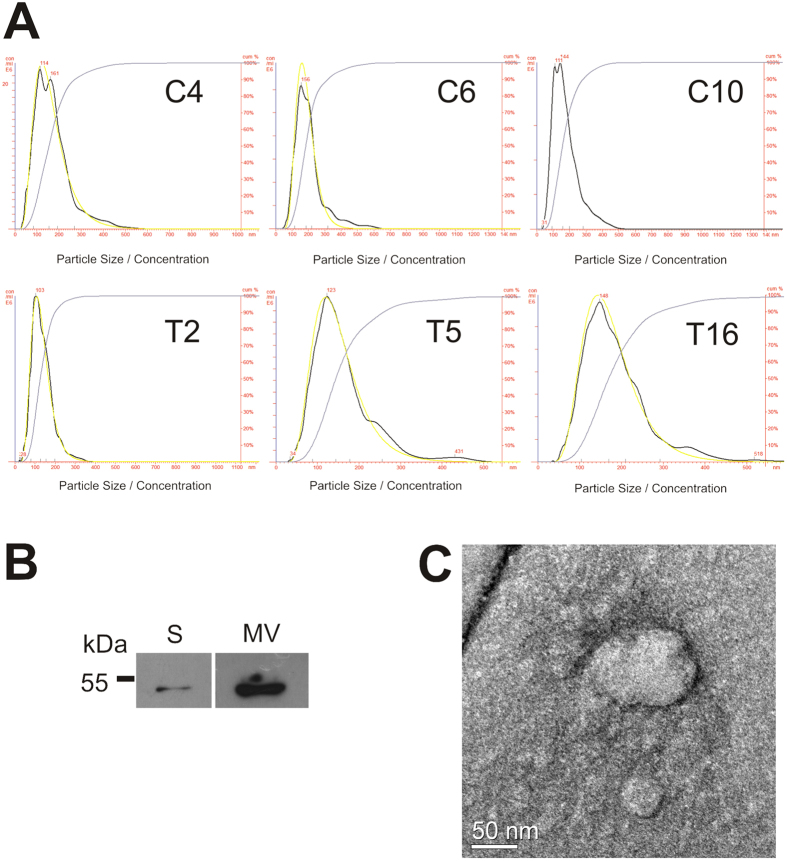
Extracellular vesicles isolated from saliva. Extracellular vesicles were isolated from the saliva of healthy and oral cancer patients using ultracentrifugation. (**A**) Nanoparticle tracking analysis of the extracellular vesicles isolated from healthy individuals (C4, C6, C10) and oral cancer patients (T2, T5, T16); (**B**) Immunochemical detection of the protein flotilin-1, a protein marker of extracellular vesicles, in the whole saliva proteome (S) and in the proteome of extracellular vesicles (EV); (**C**) Transmission electron microscopy of isolated extracellular vesicles using a negative staining method.

**Figure 5:**
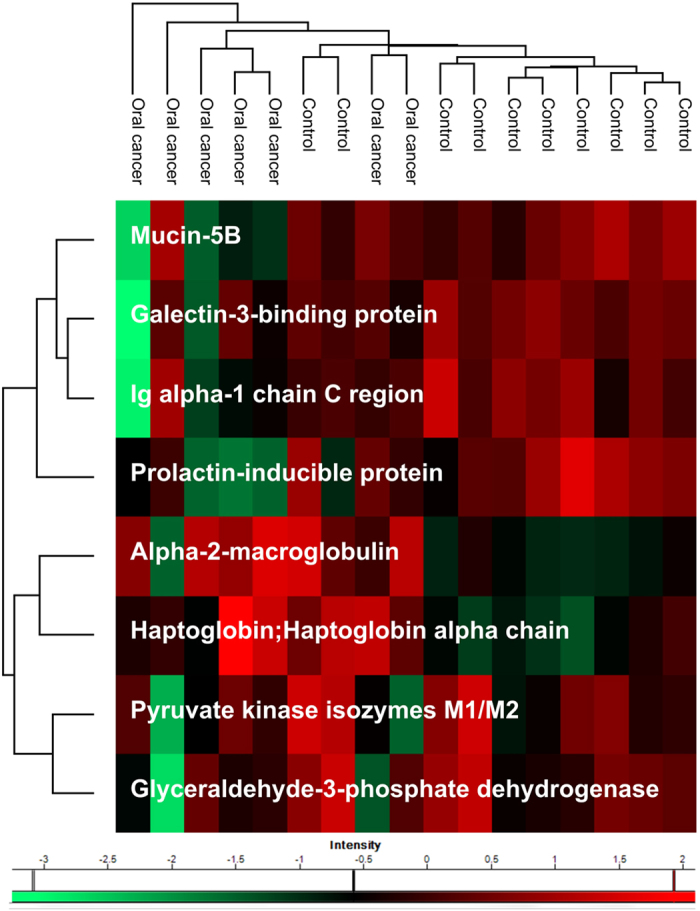
Proteins from extracellular vesicles with a significant differential abundance between healthy and oral cancer groups. Extracellular vesicles were isolated from the saliva of healthy and oral cancer individuals, and the EV protein content was analyzed by shotgun proteomics. Proteins with a significant differential abundance between healthy and oral cancer groups were identified using the label-free quantification (LFQ) method (ANOVA, p < 0.05). The Euclidean distance clustering method was used to visualize the variations in the protein abundance between the samples and is shown as normalized z-score log2 LFQ values in a heat map.

The size distribution of salivary EV samples was measured using the NTA, revealing the presence of EVs with an average size of 177.19 nm (SD = 17.22 nm) (Fig. 4A). Western blotting analysis for flotilin-1 indicated a greater abundance of this marker protein in the isolates of EVs (Fig. 4B). Furthermore, the structure of isolated EVs was observed by transmission electron microscopy (TEM-HR), which revealed that the EVs were intact after isolation (Fig. 4C). A total of 381 proteins were identified by mass spectrometry in the EV proteome from the two groups of individuals (healthy (n = 10) and oral cancer (n = 7)) (Supplemental Table 6). Considering proteins that showed a minimum of three valid expression values in at least one group, 139 proteins were subjected to statistical analysis. Eight proteins from the EVs were differentially expressed (ANOVA, p < 0.05) between the two groups (Fig. 5). Among the total 381 proteins identified, 18 proteins were exclusively detected in the healthy group and 4 proteins were exclusively identified in the oral cancer group, considering proteins identified in at least two individuals. GO enrichment analysis revealed that the biological processes “antigen binding,” “enzyme inhibitor” and “endopeptidase regulatory functions” were overrepresented in the EVs proteome (Supplemental Figure 4, Supplemental Table 7).

### Proteomics data revealed prognosis-associated proteins and functional connections among protein-coding genes and drugs

**Figure 6:**
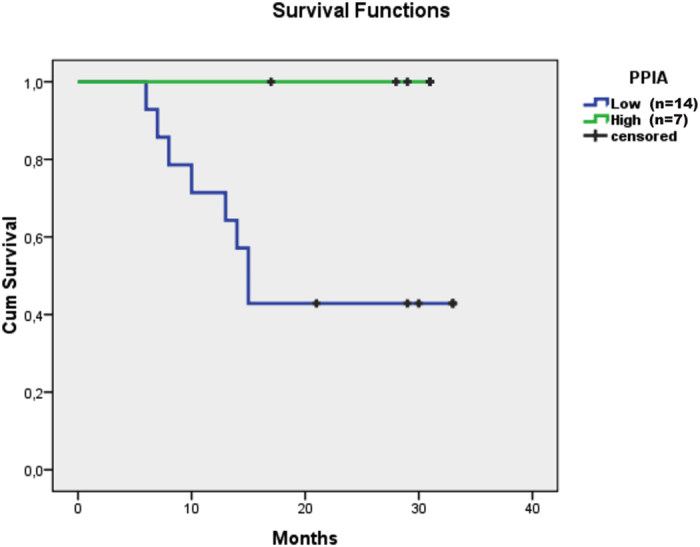
PPIA survival function from lifespan data. Kaplan-Meier curves demonstrate that patients with a PPIA low profile succumbed to the disease much earlier than those with a high profile. After 1.5 years, approximately 40% of the low profile patients survived, but all subjects in the high intensity group were alive at follow-up. A log-rank test shows significant differences between pairs, with p-value < 0.05. The number of individuals included in the analysis is shown in the figure legend.

The intensity values of the differentially expressed proteins identified in our study were used to analyze survival time. We observed that among the differentially expressed proteins, only the protein peptidyl-prolyl cis-trans isomerase A (PPIA, also known as cyclophilin-A) was statistically significant in the analysis of the mean survival time (Fig. 6). Notably, reduced abundance of PPIA appears to be a factor that may predict poor prognosis of OSCC patients. Upon observing that differentially expressed proteins may indeed possess prognostic value for patients with OSCC, we further investigated functional connections associated with these proteins.

Using the cMAP database^21^, eight compounds were identified as significantly related to alterations in the expression of the genes that code for the differentially expressed proteins (Supplemental Table 8). The small compounds terazosin, 15-delta prostaglandin J2, PNU-020031, acyclovir, dextromethorphan, yohimbic acid, benzthiazide and pseudopelletierine were ranked as the most significant compounds, with p-values < 0.01.

Furthermore, a cross-cancer analysis of the frequency of genetic alterations (mutation, deletion, amplification and multiple alterations) in the genes that code for the differentially expressed proteins in the saliva of OSCC patients indicated that in general, mutations in the genes coding for the differentially expressed proteins occur in less than 30% of the cases of head and neck cancer in datasets reported through the cBioPortal for cancer genomics (Supplemental Figure 5a). The frequency of alterations in the genes coding for proteins reported as classificatory for OSCC through the feature selection method is less than 15% (Supplemental Figure 5b) according to the information available from cBioPortal.

Furthermore, S-score (www.bioinformatics-brazil.org/S-score/) classification analysis of the list of differentially expressed proteins and proteins that were robust for classification of OSCC indicated that depending on the types of cancer compared, the same proteins may play roles as tumor oncogenes or tumor suppressors. The S-score calculation revealed that the genes coding for the differentially expressed proteins in the salivary proteome of OSCC patients display expression patterns that are most similar to the gene expression profile of the ovarian cancer dataset (Supplemental Figure 6).

## Discussion

The shotgun proteome analysis of saliva from healthy and oral cancer individuals revealed the identity of hundreds of proteins that support previous studies of the intricate effects of immune-response mechanisms in tumor development. Statistical analysis of the proteomics data revealed the modulation of expression levels of proteins related to networks and pathways that control immune responses in patients with oral cancer (Fig. 2).

Jessie and colleagues have explored the aberrant expression of proteins in the saliva of OSCC patients^15^, and the proteins serotransferrin, hemopexin, alpha-1beta-glycoprotein, alpha-1-antitrypsin, haptoglobin, fibrinogen β, complement C3 and transthyretin were detected in the saliva of OSCC patients^15^, in agreement with our proteomics results presented here. Of note, Jessie performed a very similar protein extraction procedure to that used in our present study, which is important for the establishment of a panel of biomarkers in biofluids. Haptoglobin and alpha-1-antitrypsin have previously been associated with OSCC in tissue samples and are known components of the acute-phase response, generally produced in the liver in response to the effects of cytokines^15^,^22^. However, studies of blood serum samples have indicated that acute-phase proteins, including complement C3 and haptoglobin, correlate with the progression of breast cancer, small cell lung carcinoma and lung adenocarcinoma^23^.

Previous proteomic analysis of human saliva from healthy individuals has revealed that biological processes associated with metabolic and regulatory pathways are overrepresented^24^, with nearly 50% of the proteins functionally associated with metabolism and energy pathways, protein metabolism and cell communication^16^. Therefore, the biological processes overrepresented in our dataset of salivary proteins from OSCC patients may indeed represent specific cellular responses and do not represent common features of the healthy saliva proteome.

Moreover, knowledge available on the cBIOPortal indicated that most protein coding genes for the differentially expressed proteins in the OSCC phenotype have a genetic mutation frequency of less than 30% in head and neck tumors. Furthermore, the results of the S-score analysis performed for the same dataset of protein coding genes (Supplemental Figure 6a) and proteins that may classify OSCC (Supplemental Figure 6b) indicated that complete agreement does not exist between the gene expression patterns of these genes in different types of tumors. No clear indication exists regarding the role of these genes in OSCC as putative oncogenes or tumor suppressors. However, the expression pattern (up- or down-regulation) of the differentially expressed protein coding genes in OSCC matched the gene expression pattern from six putative oncogenes and suppressor genes in an ovarian cancer dataset. Notably, tumorigenesis in ovarian carcinoma occurs through an epithelial-to-mesenchymal transition, in a manner similar to the tumorigenesis observed in OSCC^25^.

The high abundance of the proteins complement C3, complement factor B (CFB) and complement C4-B in the saliva of OSCC patients (Supplemental Table 2) has also previously been observed in tissue samples^16^. Complement activation has recently been shown to occur in ovarian cancer cells due to an autocrine effect that produces complement C3 protein, which leads to alterations in the tumor microenvironment by increasing the number of myeloid-derived suppressor cells and reducing the number of cytotoxic T cells that infiltrate into the tumor, which promotes tumor growth^26^. According to our signaling network analysis, complement C3 may be autoactivated and bind to the CFB protein, inducing the activation of the complement system (Supplemental Figure 7). Complement C3 has also been suggested to contribute to the secretion of prostaglandin E2 (PGE2) in aberrant immune responses during induced inflammation in mammals^27^. Furthermore, the overexpression of macrophage migration inhibitory factor (MIF) in the saliva from OSCC patients may also correlate with inflammatory responses through alterations in cell movement, adhesion, proliferation and invasion in head and neck squamous cell carcinoma^28^ via its functional role activating CD74 protein^29^. As a general activator of the immune response, MIF can overstimulate the secretion of prostaglandin E2 (PGE2) in endometriotic stromal cells^30^ and has direct and indirect effects on tissue remodeling and angiogenesis^31^.

Furthermore, the proteome of salivary EVs displays overrepresentation of proteins related to specialized vesicular structures secreted by the cells, including “membrane-bound vesicle” (corrected p-value = 5.44E-11), “extracellular organelle” (corrected p-value = 4.23E-10), “extracellular membrane-bound organelle” (corrected p-value = 4.23E-10) and “extracellular vesicular exosome” (corrected p-value = 5.43E-10). GO biological processes overrepresented in the proteome of EVs included “leukocyte migration involved in inflammatory response” (corrected p-value = 0.0011) and “chemokine biosynthetic process” (corrected p-value = 0.0031). Molecular functions related to “lipoprotein particle receptor binding” (corrected p-value = 0.0030), “peroxidase activity” (corrected p-value = 0.0032) and “oxidoreductase activity” (corrected p-value = 0.0032) are overrepresented in the proteome of EVs (Supplemental Figure 4, Supplemental Table 7). Interestingly, the proteome of EVs isolated from the OSCC patients was enriched in proteins with molecular and cellular functions related to “molecular transport,” mainly comprising transport of metals, and “cellular growth and proliferation” (Supplemental Table 9). These results indicate that salivary EVs may transport signaling molecules in the tumor microenvironment and may attract inflammatory cells to the tumor site by chemotactic mechanisms, which may be important in the subsequent immunoediting of inflammatory cells^32^. The study of the function of these molecules secreted by inflammatory cells may better explain how cancer cells can benefit from the function of the inflammatory system. Furthermore, tumor-derived EVs may be related to the formation of a pre-metastatic niche and signaling events in cell-to-cell communication^33^,^34^ through feedback loops between inflammation and tumor growth, possibly involving proteins such as STAT3 and IL-6R in the processes of cancer invasion and metastasis^35^.

The salivary proteome analysis from OSCC patients with and without lesions indicated that patients with active oral malignant lesions have a higher abundance of proteins related to a highly invasive phenotype and an immune response (Fig. 3, Supplemental Table 10). Among these proteins, we identified matrix metalloproteinase-9 (MMP-9), protein S100A9, myosin-9 (NMMHC II-a) and Rab GDP dissociation inhibitor beta (Supplemental Figure 8). Overexpression of MMP-9 has been detected in saliva of OSCC patients and has been implicated in promoting the invasive behavior of colon cancer cells^14^,^36^, pathological neovascularization and increased cell proliferation in other types of cancer^37^.

Furthermore, the protein S100A9, which functions as a heterodimer with S100A8, is related to the regulation of immune responses and increases in cell proliferation and motility in multiple cancers^38^. Overexpression of S100A9 has been shown to increase the size and volume of skin tumors^39^ and has also been related to chemo-attraction of leukocytes and macrophages, which may trigger pro-inflammatory responses in the tumor microenvironment^38^. However, more recent evidence has demonstrated that the S100A9/S100A8 heterodimer may actually function as a suppressor of acute inflammatory responses and contribute to reducing the gene expression of IL6, IL1-beta and TNF alpha^40^.

Our results indicate that PPIA protein is less abundant in the whole saliva of OSCC patients and is the top performing prognostic candidate protein; however, further validation in a larger population is ongoing. Previous studies have suggested that PPIA plays important roles in several mechanisms, including protein folding, trafficking and T-cell activation^41^.

We also observed modulation of proteins related to the production of vesicles and cytoskeleton dynamics. The high abundance of the Rab GDP dissociation inhibitor beta (Rab GDI) protein in the OSCC salivary proteome may indicate an increased need by cancer cells for the generation and secretion of EVs; Rab GDI has been suggested to be an important element in the control of vesicle trafficking^42^.

Furthermore, overexpression of the myosin-9 protein in patients with tumor lesions reinforces the suggested role of myosin-9 in cancer progression through affecting cell migration and actin cytoskeleton remodeling by interacting with the protein Arl13b^43^. Additional evidence has indicated that myosin-9 is involved in the directional migration of immune cells, including neutrophils^44^, and movement of T lymphocytes through the endothelial junctions^45^. In breast cancer cells, functional inhibition of myosin-9 results in impaired cell invasion^46^. In our study, myosin-9 was identified in 14 of the 17 EV samples, indicating that myosin-9 may be important in the regulation of the dynamics and organization of the cytoskeleton structures of the EVs secreted by normal and cancer cells.

The fact that OSCC individuals without tumor lesions still exhibit a salivary proteomic repertoire that differs from the healthy group is an indicator that the host response continues to be at least partially modulated once the individuals develop cancer or in response to the medical procedures performed in non-lesion patients.

Our results indicate that differentially expressed salivary proteins might be related to humoral immune responses, which in turn may be important for oral tumor development and indicate tumor progression. The proteome of salivary EVs also displayed the presence of proteins with functions related to an inflammatory response in addition to transport of metals and cellular growth and proliferation.

Therefore, the proteomic analysis of whole saliva from healthy individuals and OSCC patients indicated that the composition of saliva may indeed reflect the response of individuals diagnosed with oral cancer. The salivary composition of the patients diagnosed with OSCC is different from the saliva of healthy individuals, independently of the presence of the tumor itself, given that the saliva of patients without lesions also exhibited overexpression of known cancer-related proteins.

However, the mechanisms by which the saliva components and the immune cells interact to modulate the cellular responses in the cancer cells is not completely understood. An understanding of the role of extracellular vesicles in this cell-to-cell communication may reveal the mechanisms by which immune responses and inflammatory cells contribute to positively affect tumor growth.

## Methods

### Saliva collection and preparation

The study was approved by the ethics review board of the Instituto do Câncer do Estado de São Paulo (ICESP), Octavio Frias de Oliveira, ICESP, São Paulo, SP, Brazil and Plataforma Brasil, and written informed consent was obtained from all participants. The methods the present study were performed in accordance with the approved guidelines, and experimental protocols were approved by the ethics committee of the ICESP. Saliva samples were obtained voluntarily from healthy individuals (n = 10) and oral squamous cell carcinoma (OSCC) patients (n = 24), including patients who had undergone surgical resection (designated as “no lesion,” n = 10) and patients who had active oral malignant lesions (designated as “lesion,” n = 14) at the time of saliva collection. The clinical and pathological information for these oral cancer patients is summarized in Supplemental Table 1. Individuals first rinsed their mouths with 5 mL of drinking water and then harvested the saliva into a glass recipient. The saliva samples were aliquoted in 2 mL tubes and immediately frozen at −80 °C. The samples remained at −80 °C for long term storage.

### Whole saliva protein extraction

Saliva was initially centrifuged for 5 min at 1,500 × *g*, 4 °C to remove intact cells and debris. A volume of 100 μL of whole saliva was used in the protein extraction procedure, which was performed by homogenizing the whole saliva with 100 μL of urea buffer (100 mM Tris-HCl pH 7.5, 8 M urea, and 2 M thiourea) containing the cOmplete Mini Protease Inhibitor Cocktail (Roche, Auckland, New Zealand), 5 mM EDTA, 1 mM PMSF and 1 mM DTT. The samples were sonicated for 10 min and centrifuged at 10,000 × *g* for 5 min. Total protein was quantified using a Bradford assay kit (Bio-Rad, São Paulo, Brazil).

### Salivary EV isolation and protein extraction

Isolation of EVs was performed based on previously described methods^47^,^48^. Briefly, 1 mL of saliva was diluted at a proportion of 1:1 in phosphate buffered saline (PBS) containing protease inhibitors (1 mM PMSF, 5 mM EDTA, and cOmplete Mini Protease Inhibitor Cocktail) and 1 mM DTT and centrifuged at 200 × *g* for 5 min. The supernatants were centrifuged in consecutive steps of 2,000 × *g* for 10 min, 3,500 × *g* for 10 min, and 10,000 × *g* for 90 min, followed by a final ultracentrifugation step at 100,000 × *g* for 90 min. All centrifugation steps were performed at 4 °C. Isolated EVs were maintained at −80 °C in PBS for microscopic and nanoparticle tracking analysis or were suspended in 30 μL of urea buffer (50 mM Tris-HCl pH 7.5, 8 M urea, 2 M thiourea, and 1 mM DTT) for protein extraction. Samples in urea buffer were sonicated for 5 min at room temperature. Protein quantification was performed using a Bradford assay kit (Bio-Rad, São Paulo, Brazil).

### Immunoblotting detection

The presence of the EV marker flotilin-1 was verified by western blotting. Whole saliva and EV protein extracts were separated by 12.5% SDS-PAGE and transferred onto a nitrocellulose membrane (GE Healthcare, WI, USA), which was blocked in 1% BSA for 2 h at room temperature followed by incubation with the primary rabbit anti-flotilin-1 antibody (1:5000) (Sigma-Aldrich, MO, USA) and the secondary horseradish peroxidase-conjugated anti-rabbit IgG antibody (1:2000) (Sigma-Aldrich, MO, USA). Protein detection was performed by chemiluminescence using an ECL kit (Amersham Biosciences, NJ, USA).

### Transmission electron microscopy

Salivary EV preparations (1 × 10E8 particles·mL^−1^) were suspended in PBS and adsorbed onto glow positively charged (15 mA for 25 s) holey carbon coated copper grids (Ultrathin carbon film mesh 400 copper grid). Negative staining was performed with freshly prepared 2% aqueous uranyl acetate. EVs were imaged using a JEM-3010 transmission electron microscope equipped with a tungsten filament and operated at an acceleration voltage of 300 kV.

### Nanoparticle tracking analysis (NTA)

NTA measurements of the EVs were performed with a NanoSight NS300 (NanoSight, Amesbury, United Kingdom) equipped with a sample chamber with a 532-nm green laser. The samples were measured at room temperature in PBS for 60 s with camera gain adjustments appropriate for the correct focusing of the EVs. The software NTA 2.3 Build 0013 was used for data capture and analysis. Nanoparticle sizing and concentrations were determined based on the Brownian motion of the individual particles and light scattering measurements.

### Mass spectrometry analysis

Whole saliva protein (10 μg) and salivary EV protein (2 μg) extracts were reduced, alkylated, trypsin digested and desalted according to previous protocols^49^,^50^. Tryptic-digested peptides were dried in a speed-vac instrument and identified in an ETD enabled LTQ Orbitrap Velos mass spectrometer (Thermo Fisher Scientific, Bremen, Germany) connected to the EASY-nLC system (Proxeon Biosystem, West Palm Beach, FL, USA) through a Proxeon nanoelectrospray ion source in a data dependent mode. Peptides were separated by a 2–30% acetonitrile gradient in 0.1% formic acid using an analytical PicoFrit Column (20 cm × ID75 μm, 5 μm particle size, New objective) at a flow rate of 300 nl/min over 40 min. The nanoelectrospray voltage was set to 2.2 kV, and the source temperature was 275 °C. All instrument methods for the LTQ Orbitrap Velos were run in the data-dependent analysis (DDA) mode. The full scan MS spectra (*m*/*z* 300–1,600) were acquired in the Orbitrap analyzer after accumulation to a target value of 1e^6^. The resolution in the Orbitrap was set to *r* = 60,000. The 20 most intense peptide ions with charge states of ≥2 were sequentially isolated to a target value of 5,000 and fragmented in the linear ion trap by low-energy CID (normalized collision energy of 35%). The signal threshold for triggering an MS/MS event was set to 1,000 counts. Dynamic exclusion was enabled with an exclusion size list of 500, exclusion duration of 60 s, and a repeat count of 1. An activation q = 0.25 and activation time of 10 ms were used.

### Proteomics data analysis

Protein identification was performed with the Andromeda search algorithm within the MaxQuant v.1.3.0.3 software against the UniProt Human Protein Database (Release: June 2013, 88,820 sequences). The search parameters were set to a maximum of two missing cleavages, with a maximum error tolerance of 6 ppm for MS search and 0.5 Da for MS/MS search. Acetylation and Oxidation were set as variable modifications, and Carbamidomethylation was set as a fixed modification. Label-free protein quantification was performed using a previously described label-free quantification (LFQ) algorithm implemented in the MaxQuant software with a 2 min window for matching between runs and maximum 1% peptide and 1% protein FDR^51^,^52^. Protein intensity values were normalized using the LFQ algorithm available through the MaxQuant program and used to further identify differentially expressed proteins. Statistical analysis of the data was performed using Perseus v.1.2.7.4 software. Protein identification datasets were pre-processed for the exclusion of contaminant entries and reverse sequences identification and only identified by site entries. The proteome dataset corresponding to sample T22 was excluded from further statistical analysis due to its outlier behavior (modified z-score > 3) considering the number of proteins identified by mass spectrometry. LFQ intensity values were log2 transformed; the dataset was filtered by minimum valid values (unless otherwise indicated in the text, the minimum valid values filter was set to 3) in at least one group, and statistical significance was assessed by applying a one-way ANOVA method to identify differentially expressed proteins. Storey´s method^53^ (FDR < 0.05 and bootstrap estimation of λ) was further applied for q-value calculations through multiple hypothesis testing correction of calculated ANOVA p-values.

Additionally, data classification and feature selection analysis were performed using the Weka v.3 suite (http://www.cs.waikato.ac.nz). The support vector machine (SVM) method was applied using a sequential minimal optimization (SMO) algorithm (Polykernel –c250007 –E1.0) for training the SVM, using 66% dataset split as the training dataset, which includes the intensity values of the protein hits normalized by the z-score. The feature selection method Greedy Stepwise (forwards; -T -1.7976931348623157E308 -N -1) with Filtered Subset Evaluator as attribute estimator was applied for the z-score normalized protein intensity values for the dataset without any missing data. The dataset corresponding to sample T22 was excluded from this analysis due to its outlier behavior.

Functional annotation of the identified proteins was performed using IPA (Ingenuity® Systems, http://www.ingenuity.com). Significant interactions and pathways associated with datasets were determined by Fisher´s exact test. Overrepresented gene ontology (GO) functional categories were identified through the ClueGO v.2.0.6 plugin, using the hypergeometric test and p-value correction with the Benjamini-Hochberg method. Global *Homo sapiens* GO annotation (GO release 12.07.2013) was used as a background annotation database, and the significance threshold was set as 0.05. Overrepresented terms were visualized using the Cytoscape (v.2.8.3) software platform^54^. The raw files and protein target database were deposited in the Peptide Atlas data repository and can be accessed through the link http://www.peptideatlas.org/PASS/PASS00317.

### Assessment of prognostic protein candidates and functional connections associated with the identified proteins

Protein LFQ intensity values were used to calculate the odds ratio for the proteins identified with differential abundance in the saliva of OSCC patients. The odds ratio, p-values and confidence intervals were calculated, and proteins with an odds ratio p-value of lower than 0.05 were further considered in a survival analysis. The LFQ intensity values were used for the analysis of the odds ratio for the subset of differentially expressed proteins. The median LFQ intensity values were calculated and used as a threshold for dichotomizing the values into two categories associated with low or high intensity mean values using the SPSS (Statistical Package for the Social Sciences) 17 software package. Survival curves were created using the Kaplan-Meier method^55^ with comparisons by the log-rank test. Furthermore, our analysis of possible connections between the differentially expressed proteins and small bioactive molecules was performed using the connectivity map (cMAP; https://www.broadinstitute.org/cmap/) build 2 database. Salivary proteins that were up- and downregulated in OSCC patients were used as input data for the prediction of common gene expression changes that may occur in response to drugs, genes or proteins. Several drugs were predicted to influence the expression of these candidate protein coding genes.

Additionally, we performed a cross-cancer analysis of the frequency of genetic alterations (mutation, deletion, amplification and multiple alterations) in the genes that code for the differentially expressed proteins in the saliva of OSCC patients using cBioPortal (www.cbioportal.org). Moreover, protein-coding genes that corresponded to differentially expressed proteins identified through our proteomic approach and the proteins with potential for data classification were used as input data for the prediction of their roles as oncogenes or tumor suppressor genes in different types of cancer using the S-score system (www.bioinformatics-brazil.org/S-score/)^56^.

## Additional Information

**How to cite this article**: Winck, F. V. *et al.* Insights into immune responses in oral cancer through proteomic analysis of saliva and salivary extracellular vesicles. *Sci. Rep.* **5**, 16305; doi: 10.1038/srep16305 (2015).

## Supplementary Material

Supplementary Information

Supp Table 1

Supp Table 2

Supp Table 3

Supp Table 6

Supp Table 7

Supp Table 8

Supp Table 10

## Figures and Tables

**Table 1:** Proteins of whole saliva with potential for classification of oral cancer individuals were selected based on feature and attribute selection methods.

UniProt_AC	Gene names	Protein names	Differentially expressed*(Yes/No)
P01034	CST3	Cystatin-C	No
P23280	CA6	Carbonic anhydrase 6	No
P32926	DSG3	Desmoglein-3	Yes
Q02818	NUCB1	Nucleobindin-1	No
Q8TDL5	BPIFB1	BPI fold-containing family B member 1	Yes
Q9HC84	MUC5B	Mucin-5B	Yes
Q9NQ38-3	SPINK5	Serine protease inhibitor Kazal-type 5	Yes
P20591	MX1	Interferon-induced GTP-binding protein Mx1	No

^*^Proteins significantly differentially expressed in the whole salivary proteome or in EVs salivary proteome analysis.
