# Supplementary material for: Insights into immune responses in oral cancer through proteomic analysis of saliva and salivary extracellular vesicles
Source: Sci Rep. 2015 Nov 5;5:16305. doi: 10.1038/srep16305 (PMC4633731; doi:10.1038/srep16305)
Supplement: Supplementary Information [file srep16305-s1.pdf]

Supplementary information

Manuscript title: Insights into immune responses in oral cancer through proteomic analysis of saliva and salivary extracellular vesicles

Author´s list: *Flavia V. Winck*[1], *Ana Carolina Prado Ribeiro*[2], *Romênia Ramos Domingues*[1], *Liu Yi Ling*[3], *Diego Mauricio Riaño-Pachón*[3], *César Rivera*[1], *Thaís Bianca Brandão*[2], *Adriele Ferreira Gouvea*[2], *Alan Roger Santos-Silva*[4], *Ricardo D. Coletta*[4], *Adriana F. Paes Leme*[1,*]

[1] Laboratório de Espectrometria de Massas, Laboratório Nacional de Biociências, LNBio, CNPEM, Campinas, SP, Brazil

[2] Instituto do Câncer do Estado de São Paulo, Octavio Frias de Oliveira, ICESP, São Paulo, SP, Brazil

[3] Laboratório Nacional de Ciência e Tecnologia do Bioetanol, CTBE, CNPEM, Campinas, SP, Brazil.

[4] Faculdade de Odontologia de Piracicaba, Universidade Estadual de Campinas, UNICAMP, Piracicaba, SP, Brazil.

[5] Departamento de Ciencias Básicas Biomédicas, Universidad de Talca (UTALCA), Talca, Chile

[*] **Corresponding author:**
Adriana F. Paes Leme, Laboratório de Espectrometria de Massas, Laboratório Nacional de Biociências, LNBio, CNPEM, 13083-970 Campinas, Brazil. Phone: +55 19 3512-1118; Fax: +55 19 3512-1006, E-mail: adriana.paesleme@lnbio.cnpem.org.br

Running title: Saliva proteome analysis in oral cancer

Supplemental figure legends

Supplemental figure 1. Multiscatter plot of the LFQ intensities of control individuals

Supplemental figure 2. Multiscatter plot of the correlation between all samples analyzed by LC-MS/MS in the present study

Supplemental figure 3. Principal component analysis of whole saliva proteomics data

Principal component analysis scatter plot of Label-Free Quantification intensity values (Log2 transformed; no missing data) for the proteins identified by LC-MS/MS in the whole saliva of healthy and oral cancer individuals were subjected to a Principal Component Analysis (PCA) for data exploratory analysis. It was possible to observe that all healthy individuals were grouped in proximal coordinates, separating the individuals (healthy and oral cancer) into two different clusters. Only three oral cancer individuals were classified into the healthy group according to the PCA. Healthy individuals are indicated in gray squares with red dots. Empty gray squares represent oral cancer individuals.

Supplemental figure 4. Significant overrepresented GO functional categories in the proteome of salivary extracellular vesicles

All proteins identified in the proteome of extracellular vesicles were subjected to an enrichment analysis of Gene Ontology terms. Significant ($p<0.05$) overrepresented GO terms for Biological process and Cellular component are show.

Supplemental figure 5. Cross-cancer alteration summary for proteins differentially expressed and proteins with classificatory potential

The list of gene symbols from proteins differentially expressed and the list of gene symbols from the eight proteins with potential for classification of OSCC individuals were used as input data for the comparison of potential alterations (mutation, deletion, amplification or multiple alterations) that occur across different types of cancer according to the data available through the cBioPortal (http://www.cbioportal.org/public-portal/). (**A**) Cross-cancer summary of alterations in protein coding genes differentially expressed in the saliva of OSCC patients was generated and showed that gene mutations can achieve a frequency of less than 30% in head and neck tumors. (**B**) Cross-cancer summary of alterations in the dataset of proteins with potential to classify OSCC showed that gene alterations in this particular dataset can have a frequency of up to 20%.

Supplemental figure 6. S-score for candidate protein coding genes in different types of cancer

Gene symbols corresponding to the proteins differentially expressed in the whole saliva proteome analysis were used as input on the calculation of the S-score (www.bioinformatics-brazil.org/S-score/) based on the expression levels of the same genes in four different types of cancer (Glioblastoma, Breast, Ovary and Colorectal). (A) Heat map of the normalized S-score calculated for each protein coding gene identified as differentially expressed in our proteomics data. (B) Heat map of the normalized S-score calculated for the proteins identified as classificatory for OSCC through the feature selection method. Color scale indicates Z-score values. Blue color is

indicative of suppressor gene potential and purple color is indicative of oncogene potential.

Supplemental figure 7. Signaling network including the candidate proteins CFB and C3

Through the use of Ingenuity knowledge base, a network showing the participation of proteins overexpressed in the saliva from oral cancer patients, such as CFB, C3 and PPIA proteins, in the signaling network of ERK1/2 protein. Proteins filled in orange color represent proteins identified by LC-MS/MS in our study and proteins filled in white are inferred from the Ingenuity knowledge base.

Supplemental figure 8. Biological signaling pathways related to the differentially expressed proteins of oral cancer patients with or without lesion indicate paths for highly invasive phenotype and immune response

Protein identifiers of the proteins differentially expressed between the diagnosed oral cancer patients with and without lesion were used as input data for the inference of the significant biological pathways related to oral cancer. Proteins overexpressed in patients with lesion, including S1009A and Myosin-9, have been related to enhanced cell proliferation and collective cell migration, respectively. Furthermore, these proteins have been connected to the immune responses through macrophages-related responses.

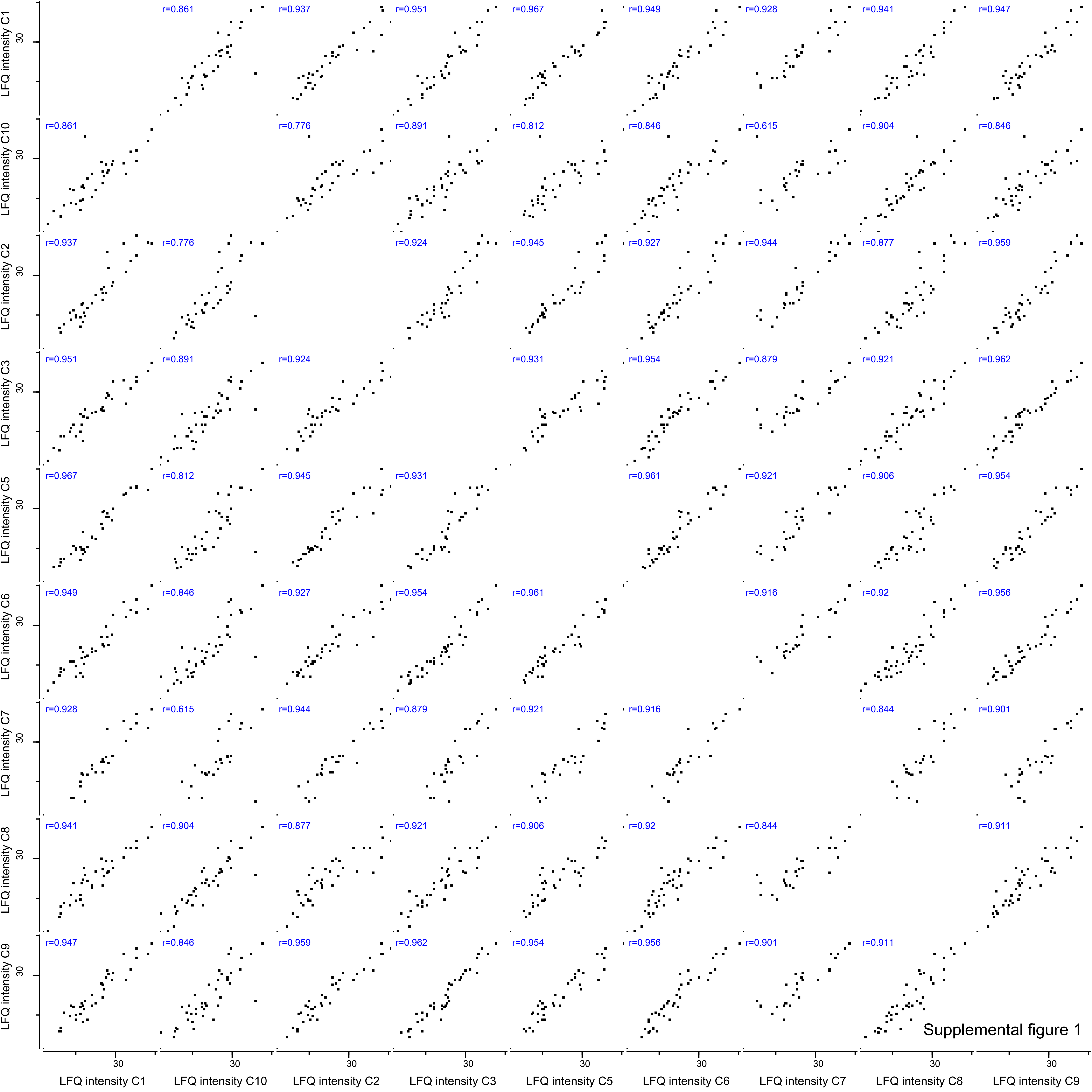

Supplemental figure 1

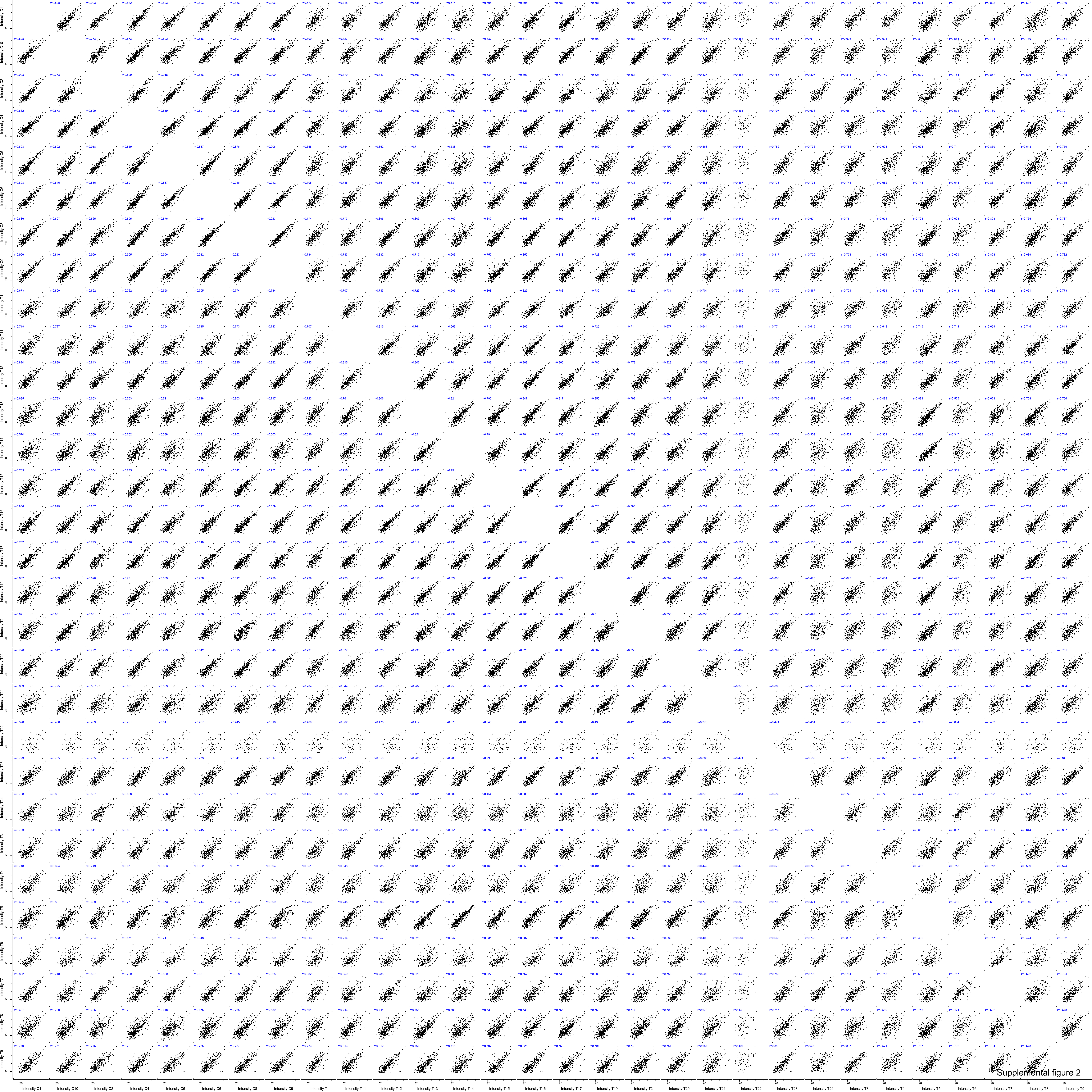

Supplemental figure 2

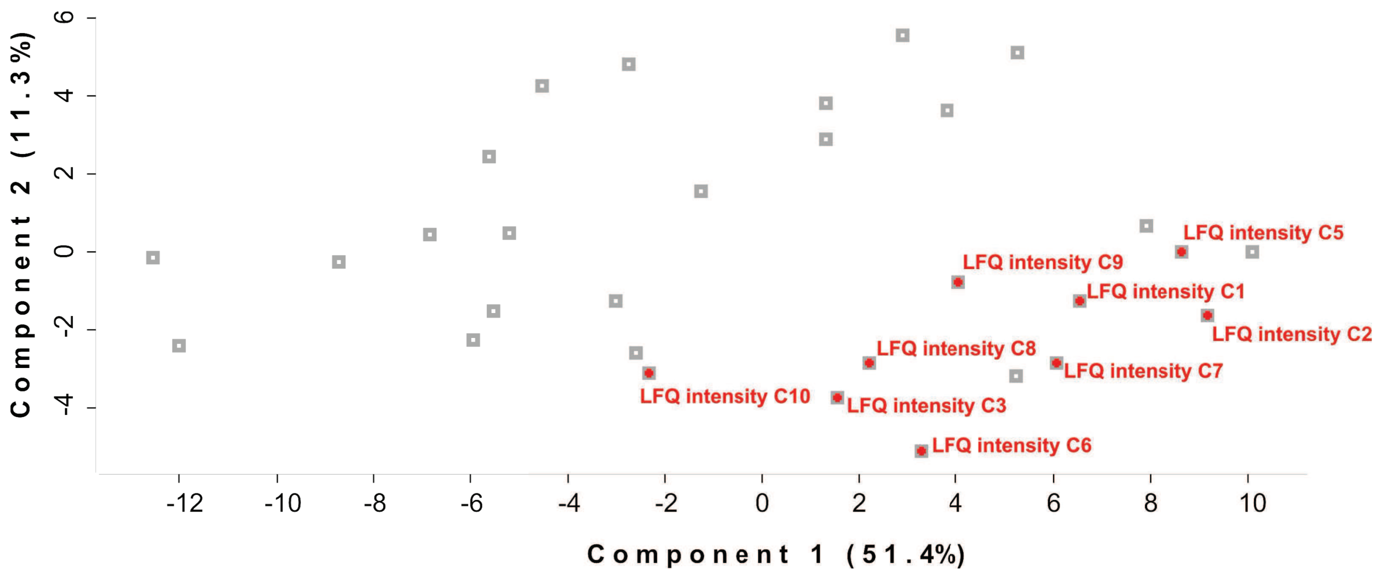

Supplemental figure 3

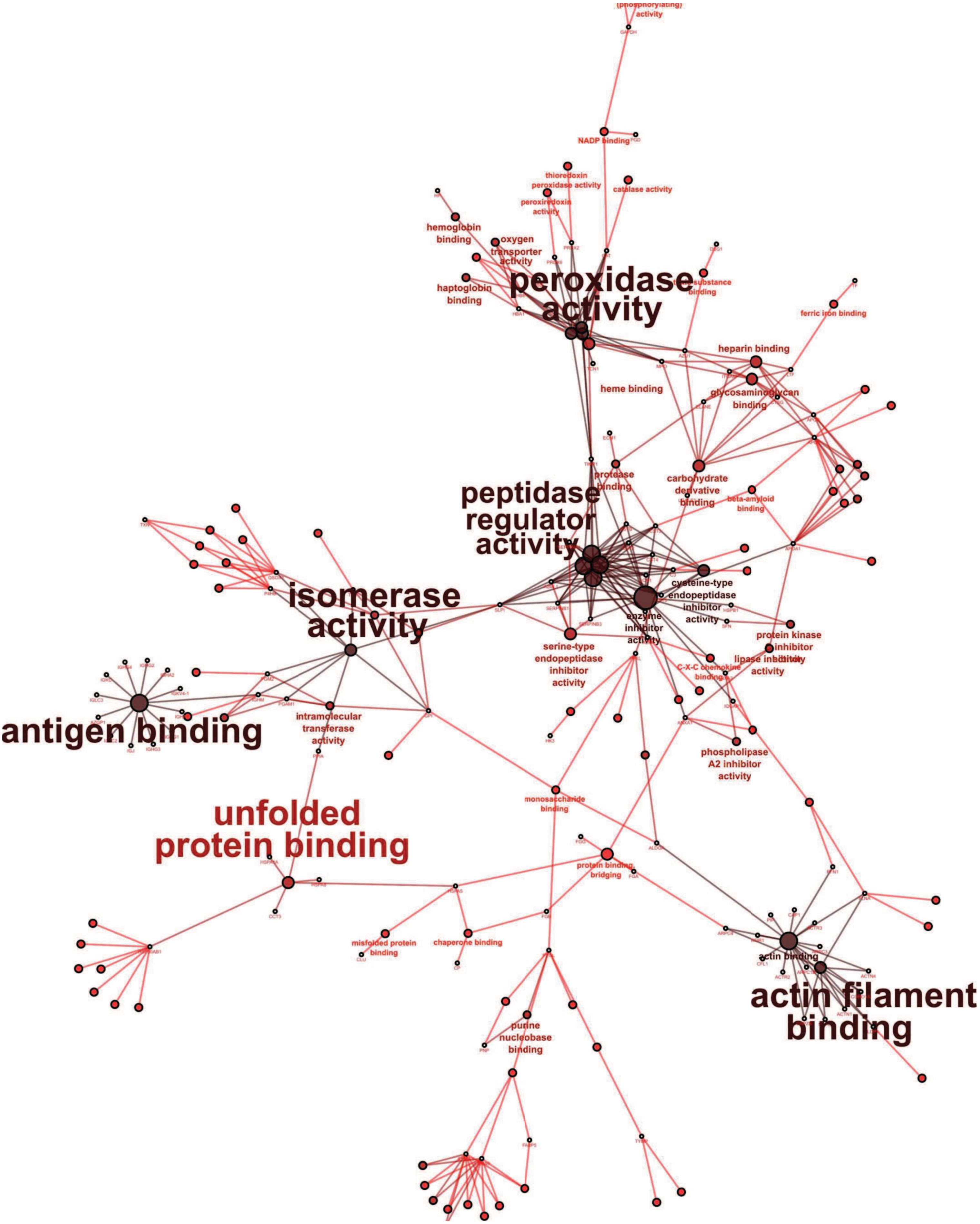

Supplemental figure 4

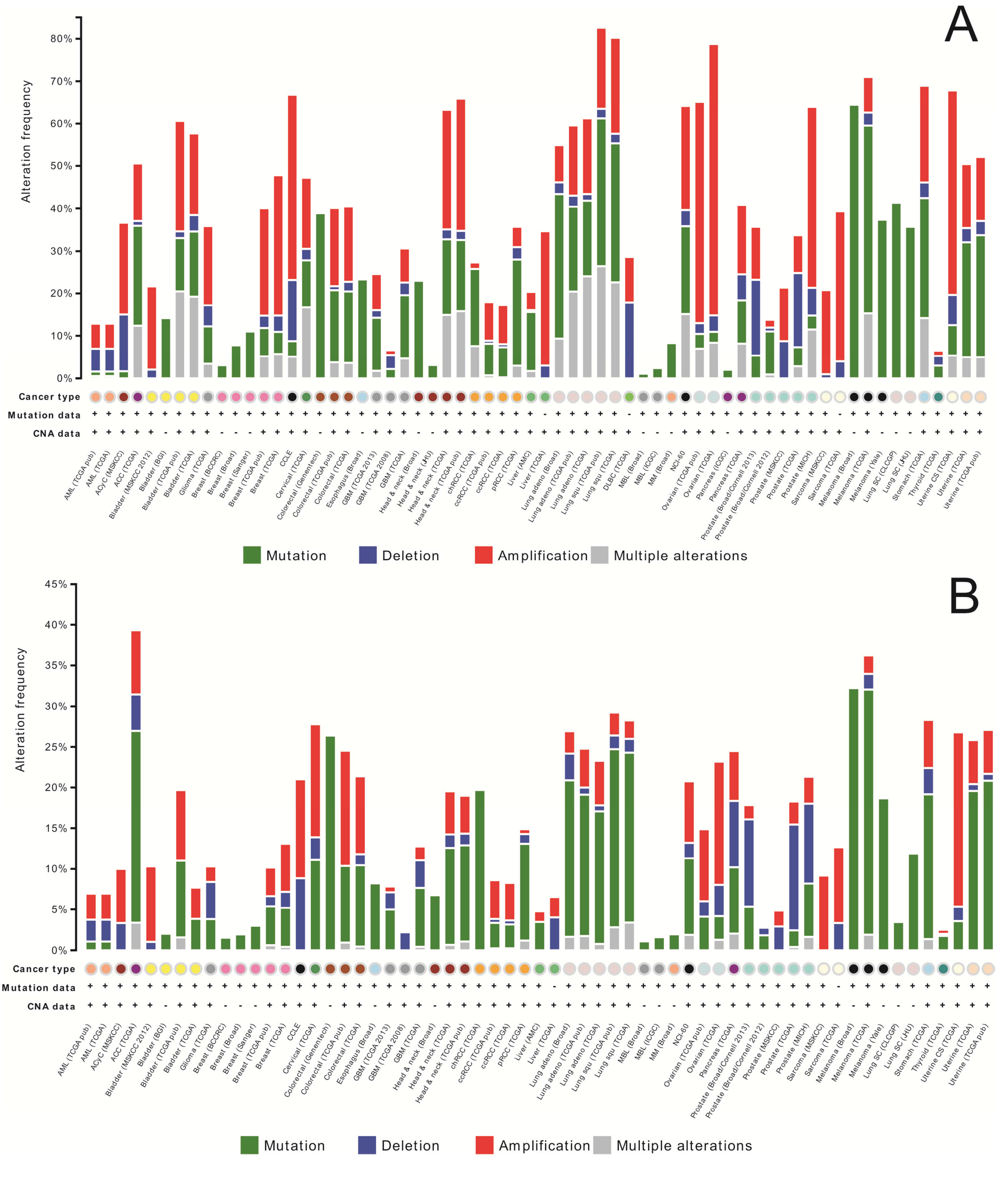

Supplemental figure 5

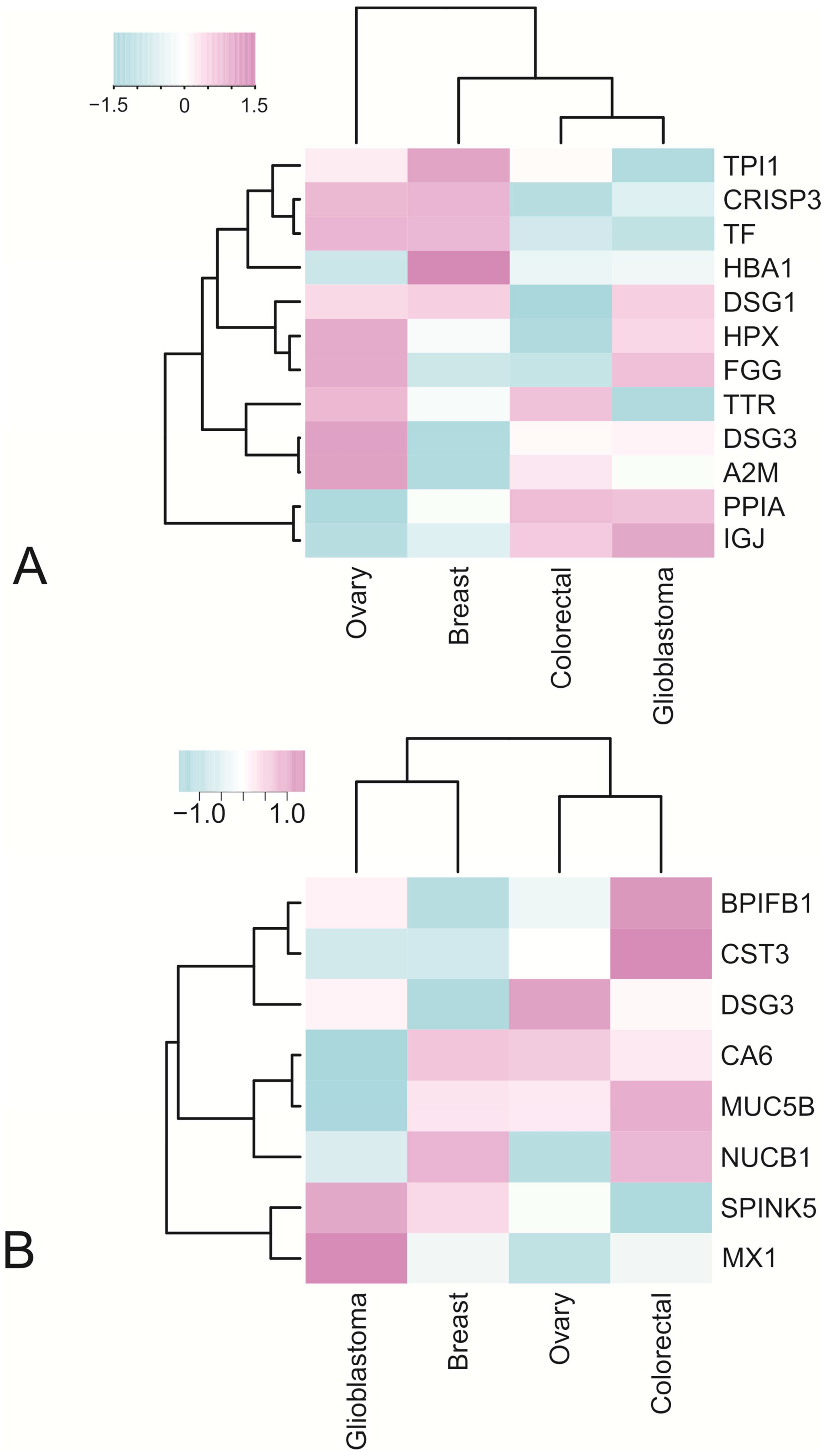

Supplemental figure 6

Path Designer Network 1

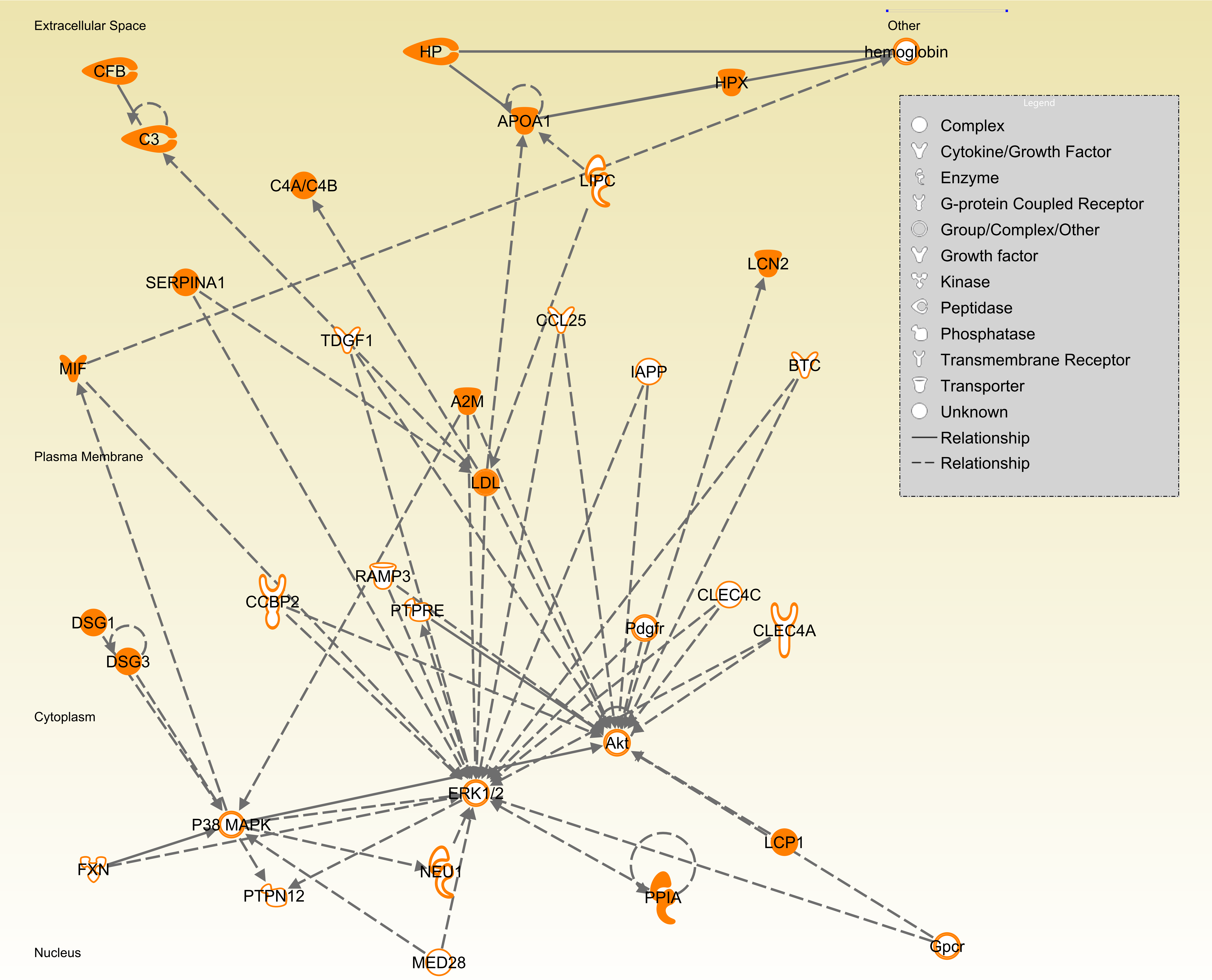

Supplemental figure 7

© 2000-2014 Ingenuity Systems, Inc. All rights reserved.

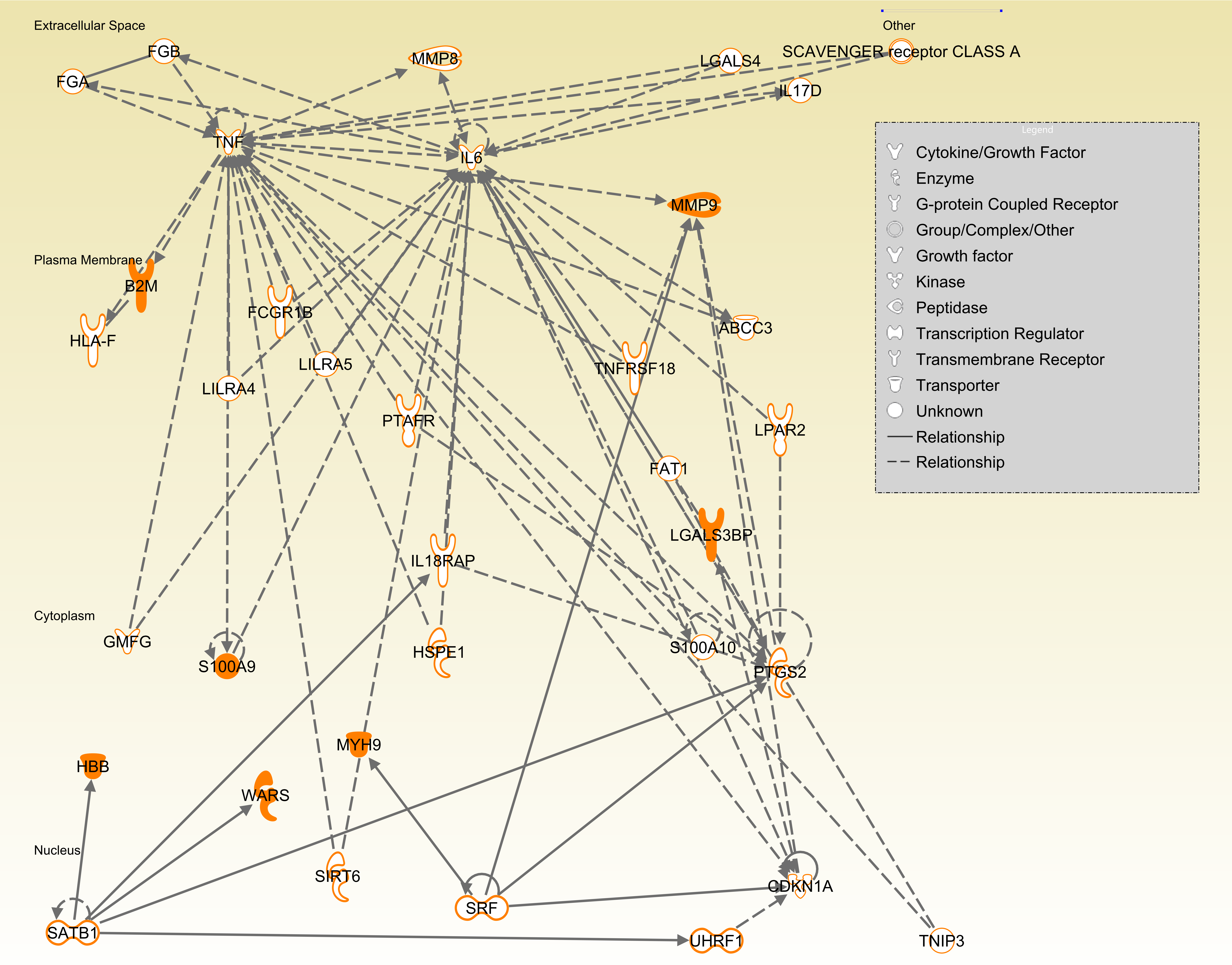

Supplemental figure 8

© 2000-2014 Ingenuity Systems, Inc. All rights reserved.

Supplemental table 4. Molecular and cellular functions overrepresented for proteins from whole saliva which are differentially expressed between healthy and oral cancer groups

Name*	p-value**	#Molecules
Cell-To-Cell Signaling and Interaction	1.09E-05- 4.84E-02	13
Cellular Compromise	1.09E-05- 4.84E-02	5
Cellular Function and Maintenance	1.09E-05 - 4.59E-02	12
Cell Morphology	1.30E-05 - 4.84E-02	4
Cellular Movement	1.31E-05 - 3.84E-02	15

***Ingenuity IPA® knowledge base annotated molecular functions.**
****Raw p-value and corrected p-value are shown, respectively.**

Supplemental table 5. Molecular and cellular functions overrepresented for proteins differentially expressed in the whole saliva of oral cancer patients with and without lesion

Name*	p-value**	#Molecules
Cellular Movement	2.41E-04 – 4.85E-02	5
Cell Death and Survival	3.01E-04 – 4.11E-02	7
Amino Acid Metabolism	6.35E-04 – 6.35E-04	1
Cell Morphology	6.35E-04 – 3.01E-02	3
Post-translational modification	6.35E-04 – 3.32E-02	3

***Ingenuity IPA® knowledge base annotated molecular functions.**
****Raw p-value and corrected p-value are shown, respectively.**

Supplemental table 9. Molecular and cellular functions overrepresented for proteins exclusively detected in the EVs from oral cancer patients

Name*	p-value**	#Molecules
Molecular Transport	1.78E-06 - 4.66E-02	23
Cellular Growth and Proliferation	8.79E-05 - 4.66E-02	35
Cell-To-Cell Signaling and Interaction	1.85E-04 - 3.56E-02	15
Free Radical Scavenging	3.32E-04 - 3.52E-02	6
Cell Signaling	4.14E-04 - 2.94E-02	8

***Ingenuity IPA® knowledge base annotated molecular functions.**
****Raw p-value and corrected p-value are shown, respectively.**
